# Is Pollution the Primary Driver of Infectious Syndemics?

**DOI:** 10.3390/pathogens13050370

**Published:** 2024-04-30

**Authors:** Merrill Singer

**Affiliations:** Anthropology, Storrs Campus, University of Connecticut, Storrs, CT 06269, USA; merrill.singer@uconn.edu

**Keywords:** particulate matter, tropospheric ozone, infectious disease syndemics, pathways of disease interaction, infectious aerosols

## Abstract

Syndemics, the adverse interaction of two or more coterminous diseases or other negative health conditions, have probably existed since human settlement, plant and animal domestication, urbanization, and the growth of social inequality beginning about 10–12,000 years ago. These dramatic changes in human social evolution significantly increased opportunities for the spread of zoonotic infectious diseases in denser human communities with increased sanitation challenges. In light of a growing body of research that indicates that anthropogenic air pollution causes numerous threats to health and is taking a far greater toll on human life and wellbeing than had been reported, this paper proposes the possibility that air pollution is now the primary driver of infectious disease syndemics. In support of this assertion, this paper reviews the growth and health impacts of air pollution, the relationship of air pollution to the development and spread of infectious diseases, and reported cases of air pollution-driven infectious disease syndemics, and presents public health recommendations for leveraging the biosocial insight of syndemic theory in responding to infectious disease.

## 1. Introduction

The spread and impact of infectious diseases in human populations were historically shaped by an assortment of factors, including the diminishment of mobile foraging, plant and animal domestication, settlement in permanent villages and subsequent urbanization, mass rural-to-urban migration, long-distance trade and mobility, war and colonialism, and significant human-induced changes in the environment, such as widespread forest clearance, dam building, and unsanitary living conditions [1]. A critical issue in health research is understanding how infectious diseases emerge, interact, and have an impact on human wellbeing. The concept of syndemics addresses these issues by drawing attention to the pathways of “adverse synergistic interaction of two or more diseases or other health conditions promoted or facilitated by social and environmental conditions” [2] (p. 7). Introduced by the author into critical health anthropology about 30 years ago, syndemic theory was influenced by the subfield’s holistic (complex system), biosocial (interactive), political–economic (structure of social relations), and ecosocial (bidirectional effect) orientation. The syndemic approach is now in use across numerous health fields, including social epidemiology, public health, medicine, nursing, dentistry, psychology, and environmental health. In the case of the latter, the focus is on the role of anthropogenic changes to the climate/environment in the formation of what are known as ecosyndemics (e.g., the contribution of deforestation and colonialism to the transition and zoonotic spread of SIV/HIV and the emergence of AIDS syndemics).

The growing body of literature on syndemics [3,4,5,6,7,8,9] highlights three key points: (1) diseases are not evenly dispersed across populations but rather tend to cluster in disadvantaged groups that suffer elevated rates of multimorbidity; (2) such clustering increases opportunities for disease interactions across various biological and psychological pathways; and (3) macro/micro-level contextual factors, including anthropogenic environmental disruptions, facilitate interaction and increase overall vulnerability and disease burden [10]. Syndemics can involve all types of disease, including both infectious and non-infectious maladies and their adverse interactions. While usually driven by social forces, including inequality, limited access to health care, and marginalization, oftentimes, oppressive social relationships are mediated by environmental factors, especially pollution. Existing research, for example, indicates that various kinds of air pollution, such as ground-level (tropospheric) ozone and particulate matter, can adversely affect proper immune functioning, contributing to breakouts of infectious diseases.

Infectious diseases, especially those that downgrade or disrupt the immune system, or involve pathogen–pathogen interaction, tend to be syndemogenic. This term refers to diseases that frequently interact with other diseases to produce adverse syndemic outcomes. Disrupted and polluted environments are associated with the emergence of new infectious diseases that afflict humans such as Zika, HIV/AIDS, and COVID-19, as well as with the resurgence of older infectious diseases like malaria, cholera, tuberculosis, yellow fever, plague, rabies, and dengue fever [11]. Environmentally promoted vulnerability to infection and the spread and pathogenicity of infectious diseases create the conditions for ecosyndemics involving at least one infectious condition and other infectious and/or non-infectious diseases. In light of a growing body of research that indicates air pollution is taking a far greater toll on human life and wellbeing than had been reported, this paper proposes the possibility that anthropogenic air pollution is now the primary driver of infectious disease syndemics.

## 2. Air Pollution and Health

Pollution, which is defined as unwanted waste of human origin that is released into the air, on land, and into the planet’s water systems, presents a clear and urgent existential threat to human health. Air pollution, in particular, is recognized as a major global threat to health. The majority of the world’s population is exposed to levels of air pollution that are substantially above WHO Air Quality Guidelines (evidence-based recommendations of limit values for specific air pollutants) and the extent of this threat appears to be far greater than was recognized just a few years ago [12]. Using computer modeling, it is estimated that the mean global air pollution concentrations increased by 38% between 1960 and 2009, dominated by increases in China and India of about 53% and 70%, respectively [13]. According to IQAir [14], an organization that tracks air quality around the globe based on data from air quality monitoring stations and sensors, 83 of the 100 urban areas with the worst air pollution in 2023 were in India. In these cities, air pollution levels exceeded the WHO guidelines by more than ten times. Only 9% of the more than 7800 cities analyzed globally by IQAir had air quality that met WHO’s standards. In the U.S., based on data from counties with monitors, air pollution declined by 27% from 2009 to 2016, but then increased by almost 6% between 2016 and 2018, a jump driven by increases in economic activity, a rise in the number and range of wildfires, and decreases in enforcement of the Clean Air Act [15].

The Lancet Commission on Pollution and Health [16] reported that pollution was responsible for 9 million premature deaths in 2015, already making it the world’s gravest environmental risk for disease and premature death at the time. Despite some global reductions in household and water pollution, as well as unsanitary living conditions, using new data from the Global Burden of Diseases, Injuries, and Risk Factors Study, the Commission [17] now estimates that pollution remains responsible for approximately 9 million deaths per year. This means that pollution accounts for one in six deaths worldwide. This is the case because the reductions that have occurred in some pollutants were offset by increased outdoor air pollution and toxic chemical pollution. The number of deaths attributable to air pollution has increased by 55% since 2000. Over 7.3 billion people globally are directly exposed to unsafe air pollution. The effects of pollution are particularly acute in low-income and middle-income countries, and in poor areas of high-income countries, where, because of environmental inequality, pollution is most severe [18,19]. At least 80% of people exposed to high levels of air pollution live in low- and middle-income countries [20], although it is estimated that only 0.001% of the world’s population breathes clean outdoor air based on WHO standards [21].

Air pollutants consist of primary and secondary components. Primary pollutants are created and released from identifiable sources (e.g., vehicles, manufacturing plants) and include fine particulate matter (a suite of pollutants varying in size and chemical composition, especially PM_2.5_), carbon monoxide (CO), nitrogen oxide (NO_2_), nitric oxide (NO), and sulfur oxide (SO_3_). Secondary pollutants form during chemical reactions among primary pollutants that occur in the lower atmosphere surrounding Earth and include ground-level ozone and aerosols from the burning of fossil fuels.

Evidence that breathing air pollutants increases vulnerability to infection dates back to at least the killer 1952 London smog, which caused the premature mortality of at least 12,000 people [22]. There is growing concern that the increases in exposure to air pollution in recent decades [23] are directly and indirectly causing significant health damage, especially in the Global South [24,25]. The countries of Asia and Africa collectively are the sites of over 92 percent of life years lost due to air pollution [26]. Even Global North countries, which are better studied, are reporting notable increases in recognized air pollution effects. Indeed, a recent nationwide study of more than three million residents of Denmark [27] found that long-term exposure to PM_2.5_ and NO_2_, respectively, was positively associated with the onset of more than 700 different diseases and other health conditions. These pollutants had their highest association with COPD, type 2 diabetes, and ischemic heart disease. In terms of infectious disease, the study linked air pollution to pneumonia and urinary tract infections. Other research indicates there is a connection between air pollutants and respiratory viral infections, including influenza, measles, mumps, rhinovirus, and respiratory syncytial virus infection [28,29]. Additionally, Croft et al. [30], in a study of adults from New York State, concluded that “increased rates of culture-negative pneumonia and influenza were associated with increased PM_2.5_ concentrations during the previous week”. Moreover, treatment for Hepatitis B viral infection, the leading cause of liver cirrhosis and liver cancer, may be adversely affected by air pollution [31].

These findings have triggered an expanded focus on the full extent of air pollution on infection and human health [32,33]. In no small part, this enhanced concern is with the impact of air pollutants on three issues of relevance to this assessment: damage to components of the immune system; damage to organs and body systems that facilitate infection; and the spread of infection through the adherence of infectious agents to ambient pollutant particles [21,34,35]. There is growing evidence, as well, that anthropogenic climate change is exacerbating the negative health effects of air pollution. Current climate projections suggest air pollution-attributed mortality will increase annual fatalities through wildfires and dust storms, while extreme temperatures, cloud cover, and precipitation will promote the formation of health-damaging ozone and particulate matter. The combined occurrence of elevated air pollution and extreme weather events highlights the potential for climate change to act as a health risk multiplier that magnifies adverse health outcomes linked to air pollution [36,37]. Additionally, climate change helps shape the geographical spread, distribution, and interaction of many infectious diseases [38].

## 3. Pathways: Air Pollution and Infectious Disease

### The Mechanisms Linking Air Pollution and Infection Include the Following

Air pollutants contribute to the development of a range of serious diseases across body systems.

Air pollutant exposure also damages both the airways and the immune system, creating vulnerability to pathogens.

Particulate matter functions as a vehicle for infectious aerosols, protecting pathogens in the air and transporting them more efficiently into the deeper airways of the lungs.

Inflammation and oxidative stress caused by air pollution during the early stages of infection render the course of infection more severe, leading to greater lethality.

A critical element in syndemic research involves identifying the specific pathways linking disease interactions. As noted above, one pathway in the adverse relationship between air pollution and infectious disease involves the roles of infection in both oxidative stress and inflammation. Although oxygen intake is indispensable for life processes, when the body’s cells use oxygen to generate energy, free radicals (molecules with unpaired electrons) are formed [39]. Oxidative stress is caused by an imbalance between the production and accumulation of reactive oxygen species, including free radicals, in the cells and tissues of the body and the inability to detoxify these reactive by-products. This disruption, which can be caused by air pollution exposure, can lead to cell and tissue breakdown and cause DNA damage. These changes include impairment of the epithelium cover of the airway tract, which can result in both acute and chronic obstructive airway diseases [40]. Oxidative stress is also known to increase the severity of viral infections [35].

Inflammation is a process that occurs in response to exposure to harmful stimuli, including bacterial and viral pathogens and environmental pollutants. The evolutionary function of inflammation is to eliminate the causes of cell injury and initiate the cell repair process. A longer duration of elevated exposure to air pollution, however, is associated with an exacerbated inflammatory response [41]. Air pollutants can stimulate pro-inflammatory immune responses across several types of immune cells, including enhancement of T helper lymphocyte type 2 and T helper lymphocyte type 17 adaptive immune responses, changes linked to the onset of allergies and asthma [42]. Sustained inflammation of the lung can lead to a substantial increase in the burden of acute lower respiratory infections and possibly tuberculosis [43]. Particulate matter can reach the alveolar sacs, the branches of air tubes where the lungs and the blood exchange oxygen and carbon dioxide. They can also move into the bloodstream, causing an inflammatory response that triggers and exacerbates respiratory diseases, including COVID-19 [44].

Moreover, chronic air pollution exposure may contribute to respiratory disease severity by interfering with Toll-like receptor (TLR) signaling networks that regulate inflammatory responses. Toll-like receptors, a class of proteins, hold a critical position in the body’s first line of defense against pathogens. They are involved in recognizing the presence of pathogens and the damage they exert on body cells and tissues. These proteins elicit an intracellular signaling cascade that begins the process of pathogen and infected cell elimination. Respiratory research suggests that air pollutants activate TLR signaling, resulting in a pro-inflammatory response in the lung. As part of this process, air pollutants can adversely modify the immune response to new threats (e.g., by changing the leukocyte T_H_1/T_H_2 profile or by reducing the immunological production of antiviral cytokines). Pollutant-driven alterations in TLR recognition of pathogens may contribute to the increased susceptibility and severity of viral infections, as well as the pathogenesis of airway diseases like asthma. [45]. Such exposure contributes to the prevalence of comorbidities associated with higher mortality and mortality [41,46].

## 4. Syndemics of Air Pollution

Syndemics of air pollution develop when inhaling polluted air damages tissue and immune function, precipitating the onset, clustering, and interaction of a wide array of infectious and non-infectious diseases. As de la Torre and colleagues [47] emphasize, “Air pollution and multimorbidity are two of the most important challenges for Public Health worldwide”. Moreover, they note, “air pollution could play a relevant role on the development and evolution of syndemics related to multimorbidity”. Research reviewed by Ciencewicki and Jaspers [34] notes an air pollution-induced enhanced susceptibility to respiratory viral infections. This suggests heightened risk for people with preexisting pulmonary diseases like asthma and COPD. These patients may have a significantly increased risk of both morbidity and mortality following infection with respiratory viruses.

In a study of COVID-19, a disease known to have its greatest impact through interaction with many pollution-linked diseases including diabetes, heart disease, and COPD, English et al. [48], examined over 3 million SARS-CoV-2 infections and almost 50,000 COVID-19 deaths. They found that compared to people living in the lowest-exposure neighborhoods, those living in neighborhoods with the highest levels of small particulate matter air pollution exposure had a 20% higher risk of SARS-CoV-2 infection and over a 50% higher risk of COVID-19 mortality. People living in the highest-exposure areas were more likely to be Hispanic and more vulnerable economically based on the widely used Area Deprivation Index. Adami et al. [49] have shown that long-term exposure to air pollution is associated with a higher risk of developing autoimmune diseases, in particular rheumatoid arthritis and connective tissue disease. People with autoimmune rheumatic, musculoskeletal, and connective tissue diseases also have been found to have greater susceptibility to COVID-19 and worse outcomes, including a higher mortality rate than those without these diseases [50]. Consequently, Nikiphorou and co-workers [51] advocate the use of a syndemic approach in the study of COVID-19 and chronic musculoskeletal conditions. Other research suggests that COVID-19 causes stem cells to produce a greater abundance of white blood cells that send inflammatory signals than the comparable stem cells of people without COVID-19. These changes suggest why this disease damages so many different organs and why some people with long COVID have high levels of body inflammation [52].

Tuberculosis (TB), a multisystemic disease most frequently caused by *Mycobacterium tuberculos*, is one of the top ten causes of death globally. Outdoor air pollution is a risk factor for pulmonary tuberculosis, the most common expression of the disease. Li et al. [53] found a potential association between outdoor exposure to various pollutants, including PM_2.5_, PM_10_, SO_2_, and NO_2_, and active TB. Similarly, You et al. [54] found that increases in the PM_2.5_ concentration during winter months were significantly correlated with a 3% jump in the number of TB cases during the following spring or summer months in both Beijing and Hong Kong. Research in India and China [55] suggests that TB infection, in turn, might play a role in the development of COVID-19 infection and in exacerbating the course of the disease for the co-infected populations. This impact appears to involve an increased abundance of circulating myeloid subpopulations of immune cells.

There are also suggested links between HIV/AIDS, another highly syndemogenic infectious disease, and air pollution. HIV and air pollution are known to be significant independent contributors to the hardening of the arteries through the buildup of plaque on the inner lining of arterial walls. A study of adolescents living in peri-urban Kampala, Uganda [56], found that the combined effects of HIV/AIDS and air pollution may amplify the development of cardiovascular disease. The researchers concluded that “early life exposure to high PM_2.5_ levels may lead to synergistic insults on cardiovascular and intestinal integrity in [HIV-infected] adolescents, compared to HIV-uninfected adolescents”. Like COVID-19, HIV can facilitate infection with *M. tuberculosis* as well as promote the transition from dormant to active infection through its impact on the immune system.

Asthma has been widely linked to ambient air pollution, including vehicular emissions. For example, research with Taiwanese middle school students found that traffic-related air pollutants, especially carbon monoxide and nitrogen oxides, are positively associated with the prevalence of asthma [57]. Moreover, there is now considerable evidence, as Murray et al. [58] (p. 376) report, that “a synergistic interaction [occurs] between allergens and viruses” in the development of asthma attacks. Note Wark and Gibson [59] (pp. 914–915), “People with asthma may frequently be exposed to more than one trigger, and these appear to interact in the development of asthma exacerbations. In experimental challenge studies, allergen responsiveness is enhanced by exposure to another trigger such as air pollution [and] viral infection”. This research suggests the occurrence of ecosyndemics involving exposure to and inhalation of air pollution in interaction with environmental allergens and pathogenic infections.

Dengue fever, a viral infection with a significant disease burden, is primarily transmitted by two mosquito species—*Aedes aegypti* and *Aedes albopictus*. The most severe form of dengue, dengue hemorrhagic fever, can cause serious bleeding, a sudden drop in blood pressure, and death. While, in many cases, people infected with dengue virus (DENV) have limited symptoms, dengue hemorrhagic fever is linked syndemically to sequential or simultaneous infection by two or more of the four viral strains of dengue (DENV-1, DENV-2, DENV-3, and DENV-4). Antibody-dependent enhancement, a process in which antibodies develop after exposure to one strain, aids in viral uptake by immune cells, resulting in the development of the severe form of dengue during infection by a different strain. Urbanization has been found to contribute to a higher risk of mosquito bites as well as to air pollution. In order to assess the impact of air pollution on mosquito activity and the incidence of dengue, Lu et al. [60] studied the disease in two cities in Taiwan. They found that particulate matter (both PM_10_ and PM_2.5_) plays significant roles in the development of dengue fever, as did climatic factors. The importance of this finding is magnified by multiple indications that dengue is rapidly spreading. While in 2000, there were approximately 500,000 reported cases and almost 20,000 deaths linked to the disease, by 2019, there were over 5 million cases and 30,000 deaths attributed to dengue [61]. While the explosion in dengue cases is generally attributed to climate change and urbanization (changes that lead to the geographic spread of mosquitos and their contact with large, concentrated human populations), the role of pollution remains to be fully explored.

Type 2 diabetes is considered one of the largest emerging health threats in the 21st century. It is estimated that there will be 380 million people with diabetes around the world by 2025 [62], and it is increasingly a health risk in the Global South [2]. Diabetes increases susceptibility to a broad range of pathogens by impacting the onset, duration, morbidity, and mortality associated with infectious disease through its role in reducing the response of T cells, diminishing neutrophil function, and downgrading humoral immunity [63]. Pulmonary tuberculosis, for example, has a recognized relationship with diabetes, and people with diabetes have a sixfold higher risk of hospitalization than non-diabetic patients during influenza epidemics [64]. Until recently, however, the contribution of infection to the onset of a highly syndemogenic disease like diabetes has received less attention. Recent data indicate, however, that the physiological changes in metabolism as a result of infection with influenza A, cytomegalovirus (CMA), and herpes simplex may trigger permanent deregulation of blood glucose levels and systemic insulin sensitivity [65,66]. To examine this connection, a population-based matched case–control cohort study in Korea recruited over 500 patients infected with cytomegalovirus, but without diabetes, and almost 3000 age/sex-matched controls that had neither condition. Participants were followed for 5 years [67]. These researchers found that the cytomegalovirus group had a much higher frequency of new-onset diabetes. Of note, the analysis showed that patients with higher CMV presence had a significantly higher diabetes incidence rate, suggesting that CMA infection increases the risk of developing diabetes. Early in the COVID-19 pandemic, as the heightened risk of severe infection for people with diabetes was becoming clear, concern was raised about the role SARS-CoV-2 might play in the onset of diabetes. There are a number of reasons for this consideration based on known infection-caused decreases in insulin secretion or increases in insulin resistance during an acute SARS-CoV-2 infection. The virus has been found in the pancreas of COVID-19 patients, suggesting it could infect pancreatic beta cells responsible for insulin production. Additionally, infection-caused inflammation could lead to both decreased insulin secretion and heightened cell resistance to insulin. The immune system produces high levels of cytokines during a severe COVID-19 infection. These proteins can cause beta cells to malfunction or perish. Research has shown a 60% increase in risk for new-onset diabetes in people suffering from COVID-19 compared with people who were never infected [68]. Recently, Rubino et al. [69], based on a growing body of literature on COVID-19-caused glucose dysregulation [70,71,72,73,74], assert “There is a bidirectional relationship between COVID-19 and diabetes. On the one hand, diabetes is associated with an increased risk of severe COVID-19. On the other hand, new-onset diabetes and severe metabolic complications of preexisting diabetes… have been observed in patients with COVID-19”.

The various studies described above highlight the link between air pollution and the onset and clustering in vulnerable populations of infectious and non-infectious diseases, and the consequent adverse disease interactions that significantly increase disease burden, especially in impoverished and Global South communities. In other words, social inequality directly drives vulnerability and the clustering and adverse interaction of infectious and non-infectious diseases in marginalized populations and significantly further drives infectious disease syndemics through environmentally mediated factors like climate change and air pollution.

## 5. Limitations

This study constitutes a preliminary but suggestive review of relevant literature across several fields of research. As such, it risks authorial bias and the influence of personal perspective. The intention of this paper is to draw attention to expanded knowledge on the multiple health effects of air population, the roles of climate change and air pollution in infectious disease syndemics, and, in the shadow of COVID-19, the growing threat of new infectious disease syndemics in the coming years.

## 6. Conclusions

This paper proposes that human populations are facing a health crisis composed of the triple jeopardy of air pollution, infectious disease-involved syndemics, and global health inequalities. The threat of this ecosyndemic continues to grow with increased global air pollution in the context of mounting climate change. Climate change is driving expansions in the ranges of infectious disease vectors like mosquitoes, ticks, sand flies, fleas, and lice. In addition, climate change is contributing to increased exposure to waterborne infectious diseases like cholera. These changes multiply the opportunity for the formation of infectious disease syndemics. As Di Ciaula et al. [75] illustrate with reference to COVID-19, “Evidence clearly indicates strong and complex links between the COVID pandemic, the global burden of noncommunicable diseases, demographic unbalance, individual vulnerability and unhealthy aging, environmental pollution, [and] socio-economic inequities…” Addressing the syndemics of air pollution requires collaboration across sectors and disciplines to better understand the complex manufacturing, transportation, land use, urbanization, and other developments that perpetuate this growing health emergency through the production of both air pollutants and climate change, and the consequent spread of emergent and re-emergent infectious diseases. Given prevailing patterns of environmental and health care injustice, societal changes to address these threats also need to be equitable. Leveraging the biosocial insight of syndemic theory necessitates the following steps:

Enhanced collaboration across disciplines and traditionally siloed fields of infectious disease, environment, climate, and social science research: The current high degree of separation among health- and environment-related disciplines is counterproductive to confronting wicked problems that, because of their complexity, defy straightforward solutions [76]. While most scientists recognize the need for interdisciplinary research, many are hesitant to abandon their narrow but familiar disciplinary focus. Addressing the case of infectious disease syndemics of COVID-19, for example, requires collaborations across the health, environment, and social science fields [35,77]. Such collaboration is needed to better understand how climate change drives the adverse health consequences of this infectious disease, who is at gravest risk, and how to change behavior to diminish exposure and reduce greenhouse gas emissions. The lack of a common language, lack of shared methodologies, lack of targeted funding, and limited experience with broad cross-disciplinary collaboration are key challenges that will need to be overcome to achieve this recommendation.

A shift in frames to forward-looking research in infectious disease medicine, environmental epidemiology, microbiology, virology, bacteriology, and related fields: As Baker et al. [78] suggest, “A changing world requires changing science to evaluate future risks from infectious disease. Future work needs to explicitly address concurrent changes: how shifting patterns of demographic, climatic and technological factors may collectively affect the risk of pathogen emergence, alterations to dynamics and global spread”. This approach is especially needed in a time of growing climate change and air pollution, emergent and re-emergent infectious diseases, mounting antibiotic resistance, and heightened armed conflict. Given our recent history, there is little doubt that new zoonotic pandemics will spread across the globe, triggering syndemic development in vulnerable populations, and that the health toll of these new diseases will be strengthened, perhaps significantly so, by air pollution.

Intensified focus on the major social, environmental, and biological conditions and biopsychological and behavioral pathways involved in infectious disease syndemics within specific place and time contexts: Such research should prioritize key factors that have significant downstream and upstream effects.

More rapid translation of environmental health research findings into innovative biosocial interventions that jointly improve global public health and health equity: Climate change, air pollution, and infectious disease syndemics are environmental justice issues. The adverse global health impact of all three of these health-related factors can be reduced simultaneously, for example, by reducing the combustion of fossil fuels and coordinating this effort to ensure that health inequalities are one of the main focuses of new policies and interventions.

At the college and university level, there is a need for increased emphasized focus on pollution and syndemics and their growing impact on human wellbeing in undergraduate education in health-related courses. Further, there is a need for training in multidisciplinary collaboration.

Action on these recommendations aligns with the UN Sustainable Development 2030 Goals, especially the objectives of ensuring clean water and sanitation, clean non-polluting energy, and climate action, as well as maximizing global collaboration. Collaboratively, researchers and clinicians have the knowledge and skills needed to drive a change in strategy, education, public health direction, and clinical practice to incorporate a syndemic perspective.
